# Predicting reactive stepping in response to perturbations by using a classification approach

**DOI:** 10.1186/s12984-020-00709-y

**Published:** 2020-07-02

**Authors:** Amber R. Emmens, Edwin H. F. van Asseldonk, Vera Prinsen, Herman van der Kooij

**Affiliations:** Department of Biomechanical Engineering, University of Twente, Drienerlolaan 5, Enschede, 7522 NB the Netherlands

**Keywords:** Standing balance, Postural control, Reactive stepping, Classification

## Abstract

**Background:**

People use various strategies to maintain balance, such as taking a reactive step or rotating the upper body. To gain insight in human balance control, it is useful to know what makes people switch from one strategy to another. In previous studies the transition from a non-stepping balance response to reactive stepping was often described by an (extended) inverted pendulum model using a limited number of features. The goal of this study is to predict whether people will take a reactive step to recover from a push and to investigate what features are most relevant for that prediction by using a data-driven approach.

**Methods:**

Ten subjects participated in an experiment in which they received forward pushes to which they had to respond naturally with or without stepping. The collected kinematic and center of pressure data were used to train several classification algorithms to predict reactive stepping. The classification algorithms that performed best were used to determine the most important features through recursive feature elimination.

**Results:**

The neural networks performed better than the other classification algorithms. The prediction accuracy depended on the length of the observation time window: the longer the allowed time between the push and the prediction, the higher the accuracy. Using a neural network with one hidden layer and eight neurons, and a feature set consisting of various kinematic and center of pressure related features, an accuracy of 0.91 was obtained for predictions made up until the moment of step leg unloading, in combination with a sensitivity of 0.79 and a specificity 0.97. The most important features were the acceleration and velocity of the center of mass, and the position of the cervical joint center.

**Conclusion:**

Using our classification-based method the occurrence of reactive stepping could be predicted with a high accuracy, higher than previous methods for predicting natural reactive stepping. The feature set used for that prediction was different from the ones reported in other step prediction studies. Given the high step prediction performance, our method has the potential to be used for triggering reactive stepping in balance controllers of bipedal robots (e.g. exoskeletons).

## Background

Human standing balance is related to the positioning and motion of the body’s Center of Mass (CoM), which can be considered as the average position of all body parts weighted according to their masses. In quiet standing, balance is maintained when the center of mass is maintained over the base of support (BoS), defined as the support polygon that is made up by the contact surfaces between the feet and the ground and the space in between. Humans use two distinct strategies to maintain standing balance without additional supports: feet-in-place strategies that do not change the BoS, and stepping strategies that do. Hence the question arises, when do people switch from a feet-in-place to a reactive stepping strategy to maintain balance? Answering this question would not only provide insight in human balance strategies, but is also relevant for human-like controller design in the fields of humanoid robotics and exoskeletons.

Which strategy humans use to maintain standing balance depends on the environmental constraints, the magnitude of a possible perturbation, and the human posture. Two feet-in-place strategies can be discerned. First, the “ankle strategy” has shown to be dominant in quiet standing [1, 2]. In this strategy, ankle joint torques are generated that result in a change in force distribution beneath the feet on the ground, and therefore in a displacement of the point of application of the net reaction force (the center of pressure (CoP)). As a result, the CoM rotates around the ankle joints. Second, the “hip strategy” is used in more challenging balancing conditions [1, 2]. The upper body is rotated through the hip joints, which results in a change in angular momentum around the CoM. The stepping strategy can be used when ankle and hip strategy are not sufficient to maintain balance, however, in reality it is also used in circumstances similar to the ones in which feet-in-place strategies are applied [3]. Hence, what exactly makes that people use a stepping strategy is unknown. Therefore, this study focuses on predicting the occurrence of natural reactive stepping.

Several methods have been proposed to detect when people take a step. Identifying this moment is key for the development of systems that support balance and/or prevent falls. These methods generally make use of an inverted pendulum model. Based on this model, in early research stability boundaries on a feasible set of CoM/CoM velocity combinations were presented, within which a movement can be terminated and a fall prevented [4, 5]. Continuing on this work, the “extrapolated center of mass position” (XCoM) - the vertical projection of the position of the CoM plus a weighted velocity term - was introduced as a stability measure [6, 7]. The XCoM indicates the position where the CoP of the inverted pendulum model should be placed to terminate movement in an upright position. Therefore, if the XCoM is within the BoS, balance can be maintained without stepping. Step predictions based on the XCoM corresponded well with the stability boundaries on the CoM and CoM velocity [4, 6]. As a follow-up on the stability boundaries, a method was presented to predict the occurrence of additional steps as well as an initial reactive step in response to a perturbation, based on the time required for the CoM to reach the boundary of the BoS [8]. A step must be taken if the “CoM-time-to-boundary” is smaller than a certain threshold. This concept was based on the idea that the central nervous system requires a minimum amount of time to initiate a step, and that a safety margin is employed that causes steps to be initiated earlier than absolutely necessary. In the field of bipedal robotics, stability measures were (independently) introduced that show a strong similarity with the XCoM, such as the “capture point” [9], the “foot placement estimator” [10], the “generalized foot placement estimator” [11] and the “maximum output admissible set” [12]. With the exception of the CoM time-to-boundary concept [8], the aforementioned models can give a good prediction of when a step *must* be taken to maintain balance. While this is useful for fall prevention, it does not necessarily predict when reactive stepping appears naturally.

Alternatively, a controller-based approach can be taken to predict balance responses and to predict the occurrence of reactive stepping. A Model Predictive Control scheme was introduced to generate ankle, hip and stepping balance recovery strategies in simulations based on a linear inverted pendulum model with a flywheel segment [13, 14]. By adjusting weights in a certain optimization criterion the different balance strategies were regulated. An advantage of such a control scheme is that it is generalizable to untested balancing conditions. However, it is unclear whether humans apply a similar optimization criterion. While step length and step duration seem to be in line with experimental results, it has not been validated whether the model can predict natural reactive stepping.

Instead of using a model-based or controller-based approach, the problem of when people take a step can also be approached as a two-class classification problem. A set of observations with known outcomes (i.e. a step is taken or no step is taken) is used to train a classification algorithm. Algorithms that are often used are discriminant analysis, support vector machines and artificial neural networks. In discriminant analysis the mean and (co)variance of each class are used to determine to which class a data sample belongs to. A decision boundary is obtained such that the distances between the class means and the decision boundary are large, while the class overlap is small [15–17]. A decision boundary can also be obtained through the application of a support vector machine. In this case not all data samples are taken into account, but only the ones closest to the decision boundary, the support vectors. The support vector machine tries to maximize the margin between the decision boundary and any of the data samples [16]. For the aforementioned classification algorithms the form of the function describing the decision boundary is known beforehand, which is not the case for artificial neural networks. The artificial neural network is a structure with an input layer, hidden layers consisting of multiple “neurons,” and an output layer. In these neurons the inputs are amplified with a weight, added together and put through a sigmoid function. The output of each hidden layer is a new input for the following hidden layer until the output layer is reached. Which classification algorithm performs best is dependent on the classification problem. In previous studies on human balance, support vector machines have been used for the detection of compensatory balance responses during walking [18], for pre-impact fall detection [19] and for predicting physiotherapists’ ratings on balance performance [20]. However, predicting natural reactive stepping is a different problem. Hence, comparing multiple classification algorithms is useful.

The performance of the classifier does not only depend on the type of classifier that is used, but it also depends on the features that are used to make a prediction. The parameters that are used for describing limits on balance without using a classification approach can be potential features for training a classification algorithm. In model-based or data-driven approaches, a common choice is to use the CoM and CoM velocity to describe standing balance [4, 5, 8, 13, 14, 21, 22]. Furthermore, the CoP and CoP velocity were used as stability margins [23–25] and the trunk angular velocity was used as a controller input for predicting balance responses [13, 14]. It is still unclear what are the best features for predicting whether people will use a stepping strategy.

In this paper our goal was to predict the natural reactive stepping response of young healthy subjects in response to an external perturbation, without using any information of the perturbation for that prediction. Here, we present a novel step prediction method that is based on binary classification. To find out which classification algorithm is most suitable for step prediction, we trained and compared several algorithms. Furthermore, we identified the key features that are best able to predict natural reactive stepping. To put the performance of our step prediction method in perspective, we compared our method to the stability boundary, the XCoM, and CoM-time-to-boundary concept.

## Experiments

**Fig. 1:**
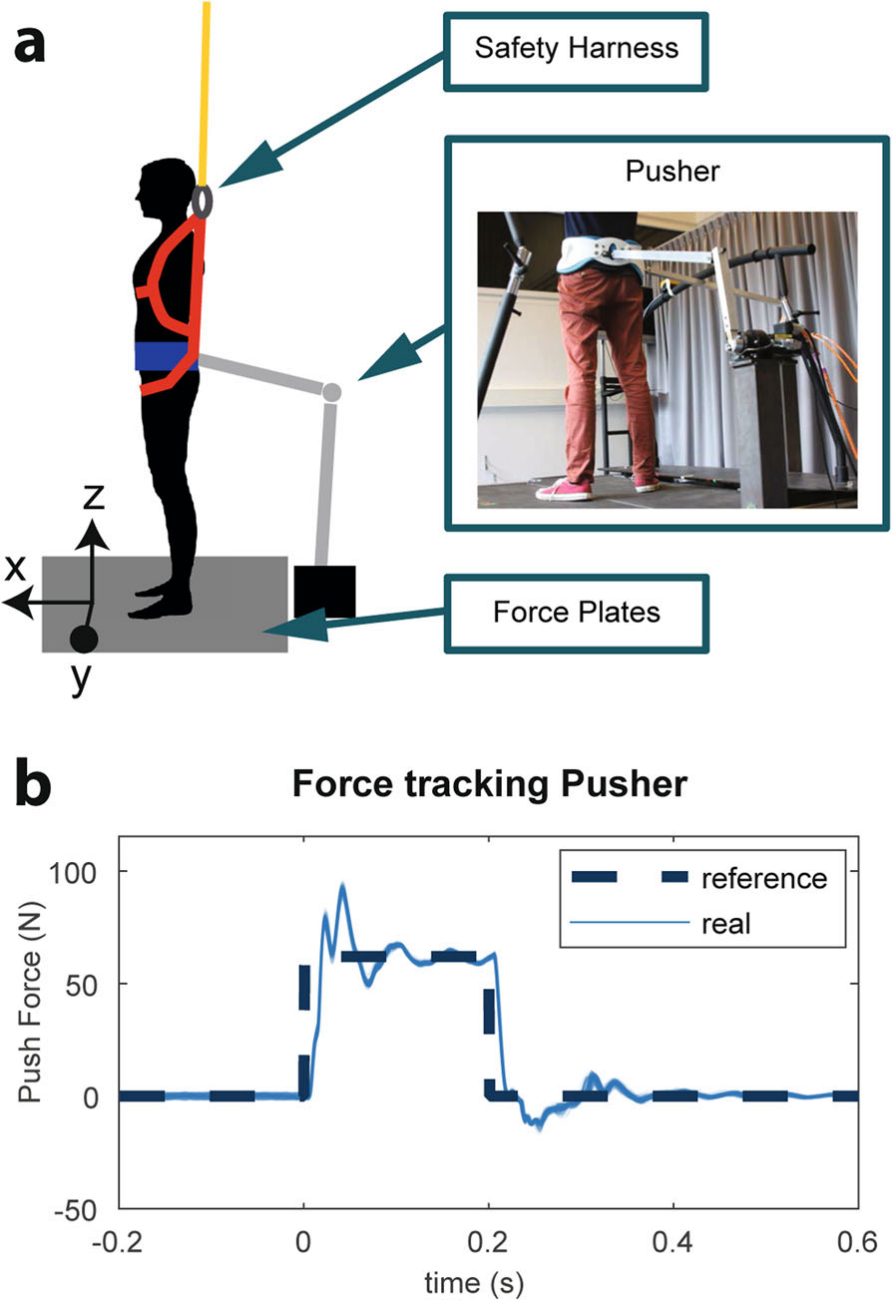
**a** Schematic overview of the experimental setup. **b** Representative reference perturbation force profile of the Pusher (reference) and all generated perturbation forces (real)

In this study, we trained several classification algorithms to predict the occurrence of natural reactive stepping. To collect training data, standing balance experiments were performed in which the aim was to mimic natural stepping in response to a perturbation. The experimental setup (Fig. 1) and study were approved by the local medical ethical committee (Medisch Ethische Toetsingscommissie Twente).

### Subjects

Ten healthy subjects (five women, age 23.6 ±1.9 years, mass 75 ±11 kg, height 1.80 ±0.09 m, mean ± SD) participated in the experiment after giving written informed consent. Subjects were only included if they had no neurological, musculoskeletal or other medical impairments that could affect functional movement performance.

### Experimental setup

Subjects stood on a custom made split-belt instrumented treadmill (Y- Mill, Motek, Amsterdam, The Netherlands) that has force plates beneath each belt to collect ground reaction forces and moments. An actuated perturbation device, referred to as “Pusher,” was placed at the rear of the treadmill to provide forward and backward perturbations at the pelvis. It consists of a motor (SMH60, Moog, Nieuw-Vennep, The Netherlands) connected to a horizontal push rod (0.8 m) through a lever arm (0.3 m), as shown on the right of Fig. 1a. At the end of the push rod a modified universal hip abduction brace (Distrac Wellcare, Hoegaarden, Belgium) was attached, which was tightly worn by the subjects. A load cell (model QLA131, FUTEK, Los Angeles, CA, USA) was mounted on the lever arm for torque sensing [26]. The rod was approximately horizontal, such that a motor torque would result in forward pushing or backward pulling of the subject. The motor was admittance controlled over Ethernet (User Datagram Protocol) at 1000 Hz, using xPC-target (The Mathworks, Natick, MA, USA). The reference perturbation force was a rectangular pulse with a duration of 0.2 s and various magnitudes. Although the Pusher motors could not track the desired reference force perfectly, the provided pushes were consistent (Fig. 1b). In between these perturbations the Pusher was transparent, meaning that the interaction force between the subject and the device was regulated to zero. Subjects wore a safety harness to prevent a fall.

### Protocol

At the start of the experiment the subject’s baseline pose was measured: a comfortable upright pose in which the feet were placed next to each other underneath the hips, with the weight evenly distributed over both legs. The positions of the feet were marked with tape on the floor and checked throughout the experiment. Subjects were instructed to maintain balance in a natural way (with or without stepping) and to return to their baseline pose within the taped area when they had taken a step. The arms were crossed in front of the body to prevent arm movements and the subjects had to look at a dedicated point that was about three meters in front of them. The subjects performed a counting backwards distraction task (in steps of 13) to enforce natural stepping.

The experiment was divided in five trials. In the first trial the goal was to find a first estimate of the stepping threshold on the perturbation force, the “force threshold,” which we defined as the strongest perturbation force for which the subject did not take a step to maintain balance. Perturbation force magnitudes were applied ranging from 0.04*mg* N to 0.16*mg* N in steps of 0.02*mg*, with a maximum of 133 N in both forward and backward directions, where *m* is the subject’s mass and *g* the gravitational acceleration. Each perturbation was repeated three times. The time between perturbations varied randomly between eight and twelve seconds.

In trials 2 to 5 perturbation forces were applied that had a magnitude close to the force threshold (0.11*mg*±0.029*mg* N in forward direction, 0.074*mg*±0.023*mg* N in backward direction, mean ± SD). Perturbation force magnitudes were applied ranging from the force threshold minus 0.02*mg* to the force threshold plus 0.06*mg* in steps of 0.005*mg* with a Pusher related maximum of 133 N for both forward and backward perturbations. Perturbation force magnitudes higher than the limit were not applied. Each force was repeated five times, resulting in a theoretical maximum total of 170 perturbations, divided over four trials. In reality this number was lower, given the limit on the perturbation force. Only the forward perturbations (722 in total) were further analyzed for predicting reactive stepping.

### Data collection and processing

Motion data were collected at 100 Hz through the use of a camera-based motion capture system (Visualeyez II, Phoenix Technologies, Burnaby, Canada). Therefore, three-LED marker clusters were placed on the subjects on the feet, lower legs, upper legs, the pelvis, the trunk and the head. In addition to the marker clusters, single LEDs were placed on the lateral epicondyles of the femur and on the lateral malleoli. Prior to the experiment, measurements were taken in which anatomical landmarks were pointed out using a LED-based probe [27, 28]. The force plate and perturbation data were collected at 1000 Hz using xPC-target (MathWorks, Natick, MA, USA). A signal composed of pseudo-random numbers and a time interval of 0.1 s between two samples was sent from the xPC-target to the Visualeyez PC and logged on both systems. By computing the cross-correlation between the (resampled) signals, the delay between the systems was established and used for data synchronization.

The motion data were reconstructed into joint and segment positions, joint angles, CoM position and velocity, and whole body angular momentum, assuming the human body segment parameters as described in Dumas et al., 2007 [28]. Because the LEDs on the marker clusters of the feet often failed during the experiment, the measurements of these clusters were unreliable. Therefore, the CoM-based parameters were reconstructed without using the position and mass of the feet. Given the low mass of the feet, and the limited movement of the feet before a step is taken, the effect of this simplification is assumed to be negligible. To be able to use the force data together with the motion data, the force data were resampled to 100 Hz. All motion and force data were smoothed with a 4th order, zero-lag Butterworth filter using a low pass cut-off frequency of 5 Hz and 8 Hz respectively. These cut-off frequencies were selected such that noise was attenuated while preserving relevant signal characteristics, based on visual inspection of the filtered and unfiltered data. The CoP was computed from the measured moments and normal forces of the force plates.

1 $$\begin{array}{@{}rcl@{}} &p_{x} &= -\frac{M_{y}}{F_{z}} \end{array} $$

2 $$\begin{array}{@{}rcl@{}} &p_{y} &= \frac{M_{x}}{F_{z}} \end{array} $$

where *p*_*x*_ is the CoP in x-direction (forward), *p*_*y*_ is the CoP in y-direction (outward), *M*_*y*_ and *M*_*x*_ are the moments around the y and x-axis respectively, and *F*_*z*_ is the vertical ground reaction force.

### Data set preparation

The balance responses were sorted in two groups: stepping and non-stepping responses. A reactive step was assumed to be taken if: 1. the vertical ground reaction force on one of the feet dropped below 20 N; 2. the step took place within two seconds after the application of the perturbation; and 3. the step length was at least 20% of the foot length of the subject. The last condition was added to prevent that voluntary foot lifts were counted as a reactive step. Since vertical foot lifts do not increase the BoS, they are not considered to be reactive stepping responses. If 1. was met, but 2. and/or 3. were not, the response was not included in further data-analysis.

During the experiment motion data of 654 stepping and non-stepping responses were recorded properly over all subjects, that is, without errors due to failing or occluded LEDs, and non-traceable marker registration issues. For these responses we checked if subjects showed consistent behavior at the onset of the applied perturbation. In particular, data were dismissed if at the onset of the perturbation: subjects showed a deviation of more than 0.03 m in forward or backward lean; subjects were swaying excessively; or subjects did not distribute their weight evenly over both legs. Furthermore, the responses in which subjects showed a lot of heel lift without taking a step were assumed to be unnatural reactions to the perturbations, as they indicate that great efforts were made not to step. These responses were therefore dismissed. A total of 580 balance responses, of which 182 stepping and 398 non-stepping, were left for step prediction. These responses were divided over three data sets: a training set, a validation set and a test set. Validation was performed using the holdout method. Because the validation set was also used for improving and selecting classifiers, an independent test set was required to verify the final performance. 15% of the stepping and non-stepping responses was assigned to the test set. Of the remaining responses, 70% was assigned to the training set and 30% to the validation set. For each subject individually the ratio of stepping and non-stepping responses was conserved over the data sets.

## Step prediction methods

**Fig. 2:**
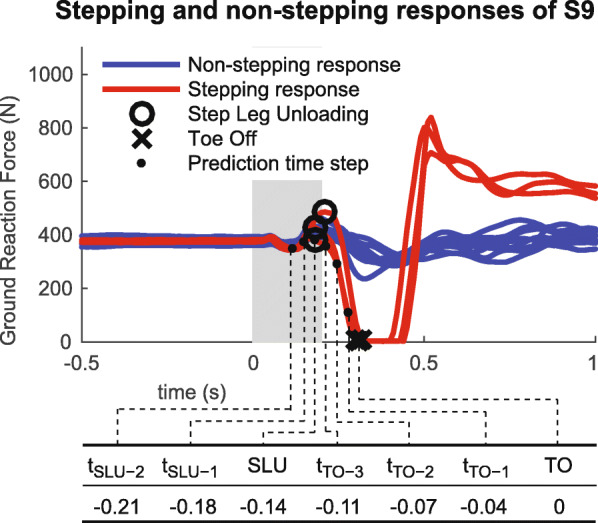
Top: Vertical ground reaction forces acting on the right foot of subject S9. The ground reaction forces were used to detect step leg unloading (circles) and toe-off (crosses) for a selection of stepping (red) and non-stepping (blue) responses. For one of the stepping responses all prediction time steps are shown (dots). The grey area represents the duration of the perturbation. Bottom: Timing of prediction time steps (in seconds) with respect to toe-off of subject S9. SLU indicates step leg unloading, TO indicates toe-off, *t*_*SLU*−*n*_ and *t*_*TO*−*n*_ indicate the *n*^*th*^ time step before step leg unloading and toe-off respectively. Note that the times are rounded to the nearest hundredth, since motion data were collected at a 100 Hz

We approached the problem of predicting whether people will take a reactive step to maintain balance as a classification problem. Hence we compared the performances of several commonly used classification algorithms: a linear discriminant analysis model (LDA), a quadratic discriminant analysis model (QDA), a linear support vector machine (LSVM) and neural networks (NN) with one hidden layer and five to eight neurons. For the training of these classification algorithms a data set of observations was needed of which the outcome classes were known. The experimental data that we collected consists of (time-series) responses that have a known outcome class instead of (time instance) observations that have a known outcome class. Therefore, we defined a relation between the outcome class of a response and the outcome class of an observation that was part of that response. For the non-stepping responses all observations from the start of the perturbation until four seconds after the start of the perturbation were labeled as “non-stepping” and included in the data set. However, for the stepping responses it did not make sense to label all observations as “stepping,” since stepping and non-stepping data were not distinguishable immediately after the perturbation was applied (Fig. 2). Therefore, we decided to include only one observation from each stepping response in the training data set. The timing and performance of the step prediction is then dependent on the chosen observation. The selection of suitable observations is further discussed in section “Data used for training.”

The performances of the trained classification algorithms were also compared to the performances of conventional step prediction methods. The classification algorithm that performed best was used to find the features that are essential for predicting reactive stepping. Finally, the step prediction performance was calculated using the best classifiers and the optimized feature sets.

### Step prediction using conventional methods

The stability boundary [4, 5], the XCoM [6] and the CoM-time-to-boundary concept [8] were used to predict the occurrence of reactive stepping. The stability boundary is a limit on the CoM velocity given the position of the CoM. For forward perturbations the limit on the anterior velocity normalized to body height is 0 *s*^−1^ when the CoM is positioned above the toe and 0.45 *s*^−1^ when the CoM is positioned above the heel [4]. We used the line passing through these points as the stability boundary. Using this method, a step was predicted if the combination of CoM position and velocity exceeded this boundary.

The XCoM is based on the inverted pendulum model of human standing balance [29, 30]. For movements in the sagittal plane it is given by

3 $$ \xi = x_{com}+\frac{\dot{x}_{com}}{\sqrt[]{\frac{g}{l}}}  $$

where *ξ* is the XCoM, *x*_*com*_ the position of the CoM in anteroposterior direction, *g* the gravitational acceleration and *l* the pendulum length. We predicted a reactive step using this method if the XCoM was larger than the upper limit of the BoS. The full size of the BoS was assumed to be the foot length from heel to toe, as measured for every subject before the experiment, but for step prediction we also used various smaller BoS sizes, since the effective BoS is smaller than the foot length [6, 7].

The CoM-time-to-boundary is the time required for the CoM to reach the boundary of the BoS, given the CoM velocity. Since we are only considering forward perturbations in the sagittal plane the instantaneous CoM-time-to-boundary (*τ*) was defined to be

4 $$ \tau = \left\{\begin{array}{ll} \frac{x_{bos}-x_{com}}{\dot{x}_{com}} & \text{if}\ \dot{x}_{com}>0 \\ \text{Inf} & \text{if}\ \dot{x}_{com} \leq 0 \end{array}\right.  $$

where *x*_*bos*_ is the upper limit of the full size BoS, that is, the position of the toes. We predicted a reactive step using this method if the CoM-time-to-boundary was smaller than a certain threshold. This threshold value was optimized such that the number of correct prediction divided by the total number of predictions was highest [8].

### Training classification algorithms

#### Data used for training

For the non-stepping responses all observations from the start of the perturbation until four seconds after the start of the perturbation were included in the data set, whereas for the stepping responses only one observation from each stepping response was included. Since reactive stepping should be predicted before a step is actually taken, we wanted to include the last observation before step initiation in the data set. However, the moment of step initiation is not unambiguously defined. A step is assumed to be taken when the foot of the stepping leg has left the ground, that is, when the ground reaction force on that foot is zero. This moment is referred to as “toe-off” (TO), as shown in Fig. 2. However, anticipatory postural adjustments may already occur several moments before TO. A typical anticipatory postural adjustment for gait initiation is peak loading of the stepping leg [31]. Although this anticipatory postural adjustment is not always present in reactive stepping [32], for the exemplary subject in Fig. 2 this moment can clearly be distinguished. Even when subjects did not show a large peak in the loading of the stepping leg, for each stepping response there was a time instant from which on the ground reaction force decreased towards zero. This moment is referred to as “step leg unloading” (SLU). Note that this moment can only be derived from the ground reaction force data when they are analyzed in reversed direction, that is, when the moment of TO is known.

**Fig. 3:**
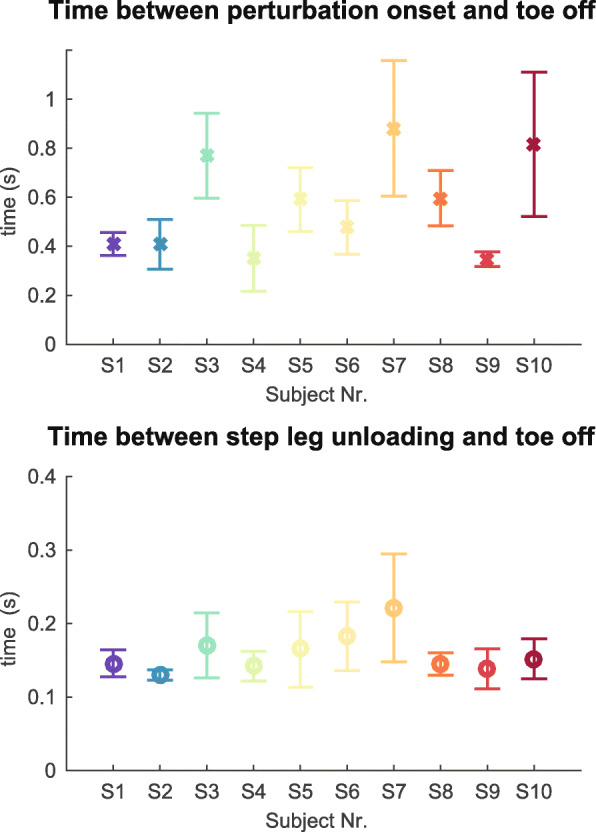
Timing of step initiation and toe-off for all subjects. Top: time between the start of the perturbation and toe-off. Bottom: time between step initiation and toe-off

Although it is reasonable to take SLU as the moment at which a step is initiated, we decided to train the classification algorithms using various observations between the start of the perturbation and to monitor the performance of the step prediction over time and to check whether good predictions can be made even before SLU. Figure 3 shows that the time between the start of the perturbation and TO varies a lot between subjects (0.56±0.20 s, mean ± SD), while the time between SLU and TO is less variable (0.16±0.03 s). Therefore, we used for every perturbation response the time between SLU and to create five equally spaced prediction time steps between and including SLU and TO, and two prediction time steps before SLU. The mean timing of these prediction time steps is shown in Table 1.

**Table 1:** Mean timing of prediction time steps (in seconds) with respect to toe-off

Prediction time step						
t _*SLU*−2_	t _*SLU*−1_	SLU	t _*TO*−3_	t _*TO*−2_	t _*TO*−1_	TO
-0.23	-0.19	-0.16	-0.12	-0.08	-0.04	0

#### Features

**Fig. 4:**
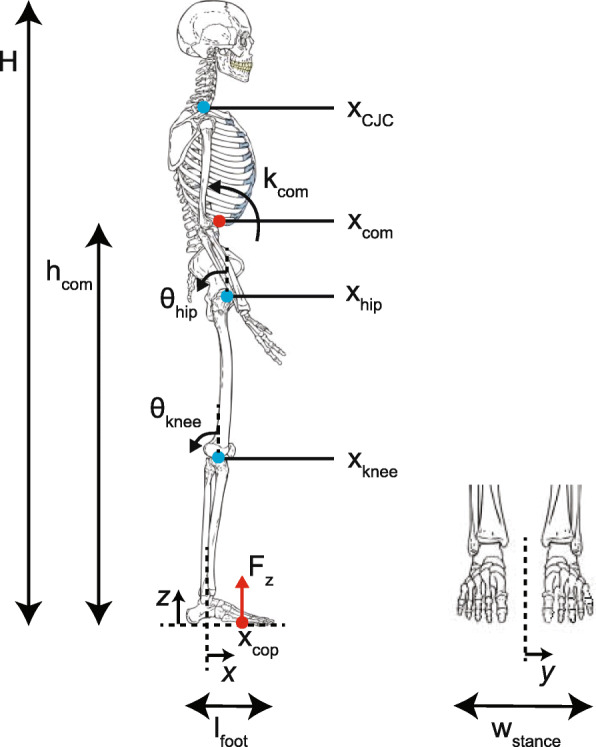
Features and normalization measures used for training the classifiers. A description of the features is presented in Table 2. Normalization measure *H* is the subject’s body height, *h*_*com*_ is the height of the center of mass in the baseline pose, *l*_*foot*_ is the length of the foot measured from heel to toe, and *w*_*stance*_ is the stance width measured from the head of the fifth metatarsal of the left foot to the head of the fifth metatarsal of the right foot. *F*_*z*_ is the vertical ground reaction force. Joint angles are positive for knee flexion and hip extension and they are defined to be zero in the baseline pose. Positions are expressed with respect to the mean position of the left and right ankle over the two seconds before the perturbation

For the training of the classification algorithms we initially used a fairly large feature set consisting of the joint positions, joint angles, CoM-based parameters and CoP-based parameters shown in Fig. 4 and Table 2. For each subject the features were normalized using the normalization factors shown in Table 2. Furthermore, features were standardized such that each feature had zero-mean and unit-variance.

5 $$ x'=\frac{x-\bar{x}}{\sigma}  $$

**Table 2:** Full feature set used for training the classification models

Feature	Description	Norm.	Mean	SD	
		factor	$\bar {x}$	*σ*	
*x*_*com*_	Position of the CoM in x-direction (anteroposterior)	1/*h*_*com*_	0.056	0.030	
*ẋ*_*com*_	Velocity of the CoM in x-direction	1/*h*_*com*_	0.000	0.057	1/*s*
*ẍ*_*com*_	Acceleration of the CoM in x-direction		0.001	0.388	*m*/*s*^2^
*k*_*com*_	Whole body angular momentum around the CoM	$1/(Mh_{com}^{2})$	0.000	0.012	*rad*/*s*
*x*_*cop*_	Position of the CoP in x-direction	1/*l*_*foot*_	0.233	0.161	
*ẋ*_*cop*_	Velocity of the CoP in x-direction	1/*l*_*foot*_	0.000	0.578	1/*s*
*ẍ*_*cop*_	Acceleration of the CoP in x-direction		0.000	2.090	*m*/*s*^2^
*y*_*cop*_	Position of the CoP in y-direction (mediolateral)	1/*w*_*stance*_	0.009	0.047	
*ẏ*_*cop*_	Velocity of the CoP in y-direction	1/*w*_*stance*_	-0.001	0.185	1/*s*
*ÿ*_*cop*_	Acceleration of the CoP in y-direction		-0.003	1.308	*m*/*s*^2^
*θ*_*hip*_	Mean joint angle of left and right hip		0.002	0.075	*rad*
*θ*_*knee*_	Mean joint angle of left and right knee		0.090	0.085	*rad*
*x*_*CJC*_	Position of the cervical joint center in x-direction	1/*H*	0.035	0.028	
*x*_*hip*_	Mean position of the left and right hip in x-direction	1/*H*	0.035	0.016	
*x*_*knee*_	Mean position of the left and right knee in x-direction	1/*H*	0.029	0.013	

Figure 4 shows the features and normalization factors. All positions are expressed with respect to the mean position of the left and right ankle over the two seconds before the perturbation. For the kinematics feature set the CoP-based features were dismissed

Where *x* is the original feature vector, $\bar {x}$ is the mean of that feature vector, and *σ* is its standard deviation. The means and standard deviations are shown in Table 2.

Since CoP data are generally difficult to collect reliably outside an experimental lab, we also trained the classifiers using a feature set that only contains kinematics.

#### Settings for training the classification algorithms

The classification algorithms were trained with the Statistics and Machine Learning Toolbox and the Neural Network Toolbox from MATLAB (2015b, The Mathworks, Natick, MA, USA) using the training set defined in section “Data set preparation.” Inputs for training the classifiers were the observations of the parameters in the feature set and their corresponding class labels. Note that the number of non-stepping observations was much larger than the number of stepping observations, since for the non-stepping responses all observations from the start of the perturbation until four seconds after the perturbation were included in the training set, whereas for the stepping responses only one observation was included. Therefore, for the LSVM, the weights on the stepping observations were empirically increased to 35 times the weights on the non-stepping observations, to enforce the classifier not to predict every new observation as a non-stepping response. The training settings for the LDA, QDA and NN were not adapted. For the LDA and QDA the training is not affected by class imbalance. The training of the NN was affected by the ratio between stepping and non-stepping responses, but this had a minor effect on the predictions made using the validation set. The effect of class imbalance on predicting the outcome classes of the validation set is discussed in “Performance of classification algorithms” section.

### Performance of classification algorithms

The classification algorithms were compared based on their performance when predicting the outcome classes of the validation set that consisted of data of 45 stepping and 103 non-stepping responses. For the stepping responses inputs for the classifiers were the observations in the time window from the onset of the perturbation up until each prediction time step, while for the non-stepping responses all observations were used. A balance response was predicted to be of the non-stepping class if all individual observations were classified as “non-stepping.” If one or more observations were classified as a step, the balance response as a whole was classified as “stepping.” Each observation was classified based on a certain “prediction score” that is the output of the classifier. This prediction score indicates the likelihood that a new observation belongs to a particular class. For the LDA and the QDA the prediction score is the posterior probability, for the LSVM it is the signed distance from the observation to the decision boundary, and for the NN it is the probability distribution over the predicted outcome classes that was generated by the Softmax output activation function [16]. Whether an observation is classified as stepping or non-stepping is dependent on the threshold on the prediction score. Generally, the predicted outcome class is the one for which the likelihood that the observation belongs to that class is highest. However, by varying the threshold on the prediction score the performance of the classifier can be optimized based on the validation set. This is desired, because we want a high performance in classifying balance responses as a whole, and not necessarily in classifying individual observations. Furthermore, by varying the threshold on the prediction score we compensate for the effects of class imbalance in the predictions of the LDA and the QDA, because changing the threshold is equivalent to changing the prior probability or the misclassification cost.

The used performance measures were the accuracy, sensitivity and specificity: the accuracy is the total number of correct predictions divided by the total number of predictions made, the sensitivity is the number of correctly predicted steps divided by the total number of steps taken, and the specificity is the number of correctly predicted non-steps divided by the total number of non-steps. Therefore, a high sensitivity means that the algorithm is highly accurate at predicting steps, while a high specificity means that the algorithm is highly accurate at predicting non-steps. By varying the threshold on the prediction score for each classifier, prediction time step and feature set the maximum achievable accuracy was obtained together with the corresponding sensitivity and specificity. If multiple thresholds lead to the same maximum accuracy, the one was used for which the sum of the sensitivity and specificity was highest. The best classifier was defined to be the classifier that resulted in the highest accuracy.

### Feature selection

Through recursive feature elimination (RFE) the goal was to find the subset of features that were most important for step prediction. Since this is a time-consuming task, only the classification algorithms with the highest performances on the validation set for prediction time step SLU were trained, as SLU is assumed to be the moment at which a subject decides to take a step. First these algorithms were trained using all features. Then, to get a measure of feature importance, they were trained using all combinations of all minus one feature. The least important feature was defined to be the feature that was absent when the highest accuracy on the validation set was achieved. This feature was removed from the feature set and the algorithms were trained again using all combinations of all minus one feature, and so on, until only one feature was left.

The best performing classification algorithms turned out to be the neural networks. Since the results of trained neural networks are dependent on randomly assigned initial weights and biases, each training run using a certain combination of features was repeated ten times. The performance on the validation set was the average performance over the ten runs. We started the RFE using the full feature set and the kinematics feature set to obtain at least two smaller feature sets: one with (possibly) CoP-based features and one with kinematic features only.

### Step prediction performance

The classifiers that performed best on the validation set for prediction time step SLU were trained using the smaller feature sets that followed from the RFE on the full feature set and on the kinematics feature set. As with the comparison of classification algorithms, the threshold on the prediction score was varied such that the highest accuracy was obtained of predicting outcome classes of the validation set. Then the performances of these optimized classifiers were computed using the test data set and compared to the performances of the classifiers trained using the full feature set and the kinematics feature set.

## Results

### Comparison of classification algorithms

**Fig. 5:**
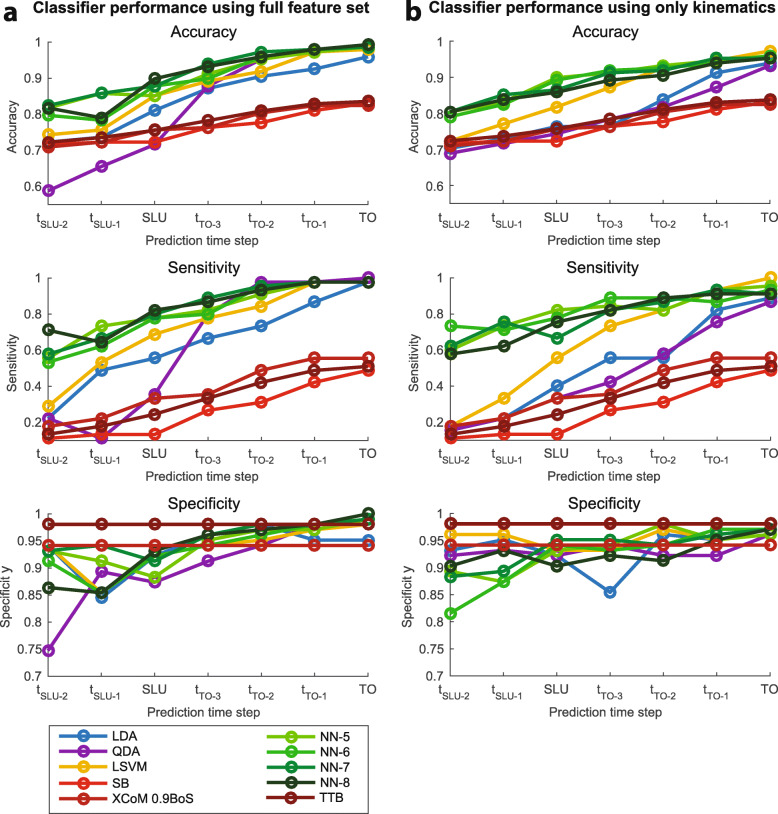
Performance of classification algorithms on the validation set using **a** the full feature set including CoP related features and **b** the kinematics feature set. In the Specificity plots lines for the CoM-time-to-boundary (TTB) and stability boundary (SB) concepts are overlapping. SLU indicates step leg unloading, TO indicates toe-off, *t*_*SLU*−*n*_ and *t*_*TO*−*n*_ indicate the *n*^*th*^ time step before step leg unloading and toe-off respectively. Predictions were made using various observation time windows: from the start of the perturbation until each prediction time step

Figure 5 shows that generally the highest accuracy on the validation data set was obtained using a NN, for both the full feature set and the kinematics feature set, independent of the observation time window. The NN models score high on the sensitivity, while the obtained specificity is similar to the other classifiers, indicating that they are able to accurately predict both stepping responses and non-stepping responses. Overall, the performance of classifiers trained using the full feature set was higher than the performance of classifiers trained using only kinematics.

The stability boundary, XCoM and CoM-time-to-boundary performed worse than the trained classification algorithms. Using the CoM-time-to-boundary or stability boundary for step prediction resulted in the highest specificity, but the matching sensitivities, and therefore the accuracies, were low. Therefore, the stability boundary and the CoM-time-to-boundary are conservative step predictors that are reluctant to predict a stepping response. The performance of the XCoM using a BoS size equal to the foot length was similar to the performance of the stability boundary and therefore not shown in Fig. 5. By adapting the size of the BoS, and therefore the upper limit on the XCoM, the sensitivity and accuracy could be increased. Hence, reducing the effective BoS increased the number of correctly predicted stepping responses. Still, the optimized XCoM model performed worse than the trained classification algorithms.

The accuracy of the prediction increased with the length of the observation time window. When predictions were made up until TO, the classifiers trained using the full feature set predicted balance responses with a maximum accuracy of 0.99, which reduced to 0.90 and 0.82 for predictions made up until SLU and the second time step before SLU (t _SLU-2_) respectively. Predictions made using the kinematics feature set were slightly worse: for predictions made up until TO, SLU and t _SLU-2_ the maximum accuracies were 0.97, 0.90 and 0.80 respectively.

For predictions made up until SLU the best performing classification algorithms were the NN with eight neurons for the full feature set and the NN with five neurons for the kinematics feature set. Therefore, these classifiers were used for RFE.

### Feature selection

**Fig. 6:**
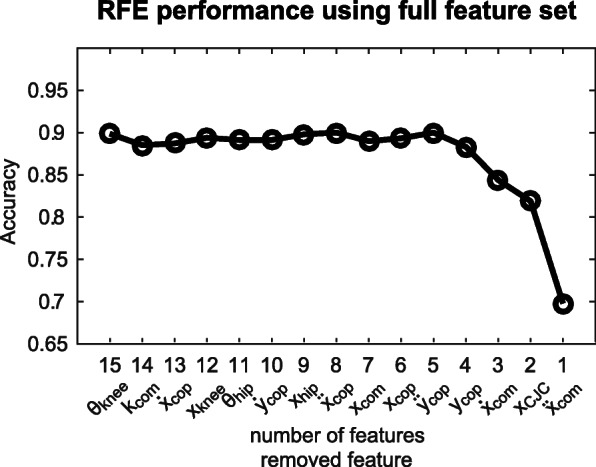
Recursive feature elimination based on the full feature set including CoP-based features using a neural network with one hidden layer and five neurons. The moment of step leg unloading was used as prediction time step. The accuracies were achieved on the validation set

**Fig. 7:**
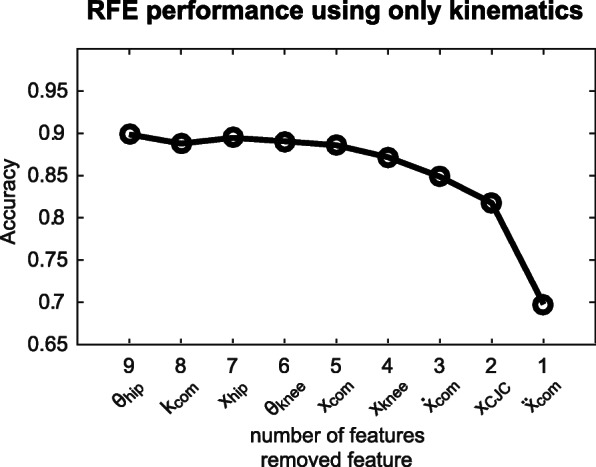
Recursive feature elimination based on the kinematics feature set using a neural network with one hidden layer and five neurons. The moment of step leg unloading was used as prediction time step. The accuracies were achieved on the validation set

When the full feature set was used for RFE the classification accuracy on the validation set remained constant for the remaining features until five features were left. These features were: the acceleration of the CoM, the position of the cervical joint center (CJC), the velocity of the CoM, the position of the CoP in mediolateral direction and the acceleration of the CoP in mediolateral direction, in order of importance (Fig. 6). As more features were removed the accuracy decreased. A similar trend was found when the kinematics feature set was used for RFE (Fig. 7). In this case the most important features were also the acceleration of the CoM, the position of the CJC and the velocity of the CoM, supplemented with the position of the knee and the position of the CoM. Based on this RFE three reduced feature sets were defined: two feature sets consisting of the five most important features following from the RFE on the full feature set and the RFE on the kinematics feature set, and one feature set consisting of the three features that both feature sets have in common.

### Test set performance

We tested the step prediction performance of five classifiers:

- a neural network with eight neurons trained with the full feature set,
- a neural network with five neurons trained with the kinematics feature set,
- a neural network with eight neurons trained with the reduced feature set including CoP-based features, that is, the feature set containing the five most important features resulting from the RFE on the full feature set,
- a neural network with five neurons trained with the reduced kinematics feature set, that is, the feature set containing the five most important features resulting from the RFE on the kinematics feature set,
- a neural network with eight neurons trained with the three most important features resulting from both the RFE on the full feature set and the RFE on the kinematics feature set.

**Fig. 8:**
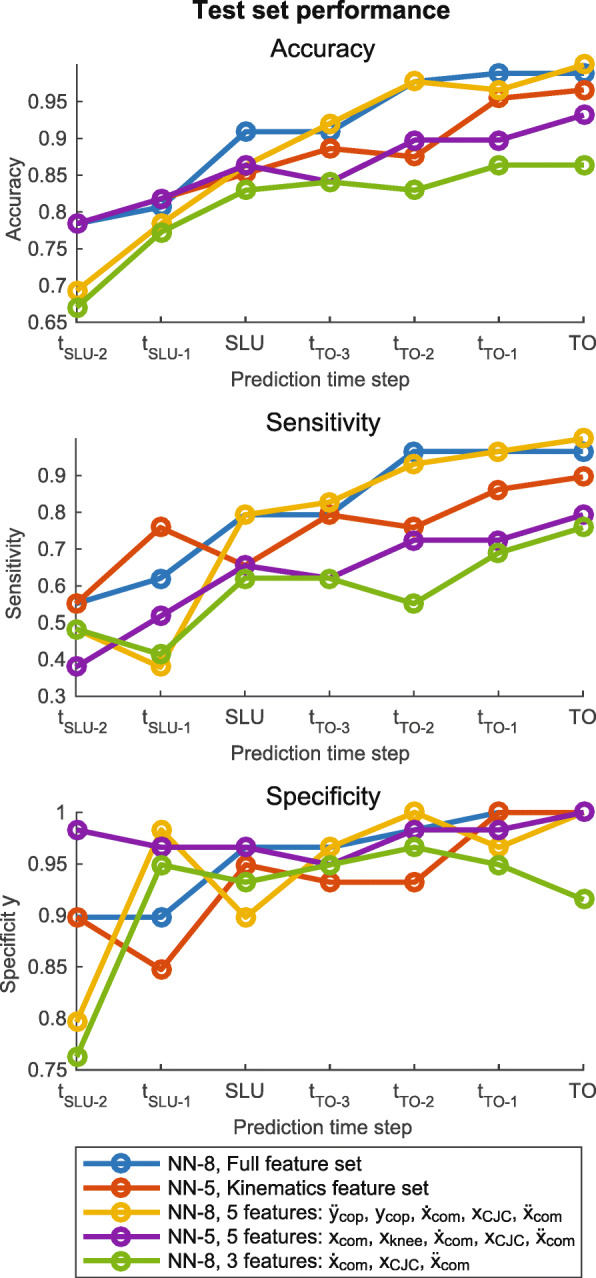
Performance on the test data set using the original full feature set and kinematics feature set, and the three reduced feature sets that followed from recursive feature elimination. SLU indicates step leg unloading, TO indicates toe-off, *t*_*SLU*−*n*_ and *t*_*TO*−*n*_ indicate the *n*^*th*^ time step before step leg unloading and toe-off respectively. Predictions were made using various observation time windows: from the start of the perturbation until each prediction time step

The shorter the observation time window, the lower the accuracies of the predictions. The corresponding sensitivities showed a similar trend, whereas the corresponding specificities were generally high for all prediction time steps (Fig. 8).

Figure 8 shows that reactive stepping can be predicted with a maximum accuracy of 1.00, 0.91, and 0.78 for predictions made up until TO, SLU, and t _SLU-2_ respectively. Using the full feature set the corresponding accuracies were 0.99, 0.91 and 0.78 (Table 3). When the reduced feature set including CoP-based features was used, accuracies similar to the ones using the full feature set were achieved for late prediction time steps, but accuracies for early predictions were low (0.69 for predictions made up until t _SLU-2_). On the contrary, using the kinematics feature set or the reduced kinematics feature set, the early prediction accuracies were similar to the ones using the full feature set, but accuracies for late prediction time steps were lower (<0.97 for predictions made up until TO). The accuracy of predictions made up until SLU using the full feature set (0.91) was clearly higher than the ones obtained using the reduced feature set including CoP-based features and the reduced kinematics feature set (0.86 for both sets). The prediction accuracies were lowest when only three features were used (0.86, 0.83 and 0.67 for prediction made up until TO, SLU and t _SLU-2_ respectively).

**Table 3:** Prediction accuracy, sensitivity, and specificity on the test set for each prediction time step obtained using a neural network with eight neurons that was trained with the full feature set

	Prediction time step						
	t _SLU-2_	t _SLU-1_	SLU	t _TO-3_	t _TO-2_	t _TO-1_	TO
Accuracy	0.78	0.81	0.91	0.91	0.98	0.99	0.99
Sensitivity	0.55	0.62	0.79	0.79	0.97	0.97	0.97
Specificity	0.90	0.90	0.97	0.97	0.98	1.00	1.00

## Discussion

In this work we predicted natural reactive stepping responses through the use of classification algorithms. By comparing various classification algorithms we found that neural networks with one hidden layer and five or eight neurons were most suitable for step prediction using an input feature set without or with CoP-related features, respectively. The step prediction accuracy was dependent on the length of the observation time window. For predictions made up until the time steps between (and including) step leg unloading and toe-off, high accuracies (>0.9) could be obtained. The most important input features were the acceleration of the CoM, the position of the cervical joint center and the velocity of the CoM. However, the performance of the classifier trained using only these three features was low (Fig. 8, “NN-8, 3 features”). When the position of the knee and the position of the CoM were added as a feature, performances similar to the ones using the full feature set could be obtained for the prediction time steps before step leg unloading (Fig. 8, “NN-5, 5 features”). For the prediction time steps after step leg unloading the position and acceleration of the CoP (in mediolateral direction) could best be added (Fig. 8, “NN-8, 5 features”).

### Feature contribution

The performances of the classifiers are dependent on the used features and the assumed relation between those features. In our classification algorithms comparison (Fig. 5) the performances of the conventional methods (stability boundary and XcoM) were low, although at TO they were similar to the ones presented by Pai et al. [5] for young healthy subjects (accuracy 0.83, specificity 1). Firstly, this is a result of the assumed (approximate) linear relation between the features. Our study showed that the trained NNs performed better than the other classifiers, especially at early prediction time steps. Using a NN with one hidden layer the decision boundary can be described by a wide variety of non-linear functions [16, 33], whereas for the other classifiers the function form is fixed (i.e. linear or quadratic). Secondly, the low performance of the conventional methods is a result of the choice to use the CoM and CoM velocity as features. Even with a linear model (LSVM) a higher accuracy could be obtained by using more features (Fig. 5), showing that using only the CoM and CoM velocity is not a good choice for the prediction of natural reactive stepping.

We found that the three most important features for predicting reactive stepping were the acceleration of the CoM, the location of the CJC and the velocity of the CoM. The velocity and acceleration of the CoM are parameters often associated with balance [34–36], but the location of the CJC is not commonly used. The positioning of the CJC is related to upper body rotation, which is assumed to play an important role in balance, for example in the ‘hip strategy’ [1]. In our feature set the position of the CJC is the only body-fixed feature in which flexion of the trunk is expressed and this feature could be a measure of whether the hip strategy can be applied effectively and efficiently to counteract the perturbation. Therefore, this result indicates that for predicting natural reactive stepping it is important to take this degree of freedom into account.

The added value of CoP-related features in the feature set is dependent on the observation time window. For predictions made up until a time step after SLU, the feature sets that contain CoP-related features performed better than the feature sets that only contain kinematics (Fig. 8). For predictions made up until TO even the maximum prediction accuracy (1.00) was obtained using the five features that resulted from the RFE on the full feature set (Fig. 8, “NN-8, 5 features”). At this time step the weight of a subject, and therefore the CoP, has fully shifted to one leg. Hence, it makes sense that good predictions can be made when the CoP in mediolateral direction is part of the feature set. However, for predictions made before SLU, the reduced feature set including CoP-based features performed worse than the kinematics feature set and similar to the feature set with only three features (Fig. 8, “NN-8, 5 features”, “NN-5 Kinematics”, and “NN8-8, 3 features” respectively). This indicates that the CoP-related features that are important predictors for predictions made up until TO are not relevant for early predictions.

### Methodological considerations

The sensitivity of the step prediction was quite low at early prediction time steps (0.38 for predictions made up until t _SLU-2_ for the reduced kinematics feature set, Fig. 8, “NN-5, 5 features”). Hence, at early prediction time steps the stepping responses are often not correctly predicted. This is a result of the choice to optimize for the accuracy. Due to the imbalance in the number of stepping responses and non-stepping responses in our data set, an incorrectly predicted stepping response has a larger influence on the sensitivity than an incorrectly predicted non-stepping response has on the specificity. However, they both have the same influence on the accuracy. In some cases it is desired to achieve a high sensitivity. For example, in fall prevention it can be particularly important that steps are predicted correctly on time, because it is better to make an unnecessary corrective step than to step too late. Using our prediction method this can be achieved by choosing the threshold on the prediction score such that a high sensitivity is obtained. However, that comes at a price of a lower accuracy.

The data collected from the perturbation responses were time series. However, we did not make the predictions based on the complete time series, but based on individual observations in the time series. For the stepping responses only one observation from each stepping response was included in the training set. Furthermore, we predicted a stepping response if at least one observation was classified as a step, that is, based on a single hit. We also trained the classification algorithms using more observations, e.g. all observations between the perturbation and the prediction time step, but that did not improve the performance. Although our initial attempts did not show any added value of including more samples, more extensive testing should be done to determine whether the prediction performance can be improved by including multiple observations from each stepping response in the training set, or by predicting a stepping response based on multiple hits.

Furthermore, it is possible that the prediction performance can be improved when the causal relation between time samples is taken into account. We tried to include information of multiple observations in one sample by passing the data through a moving average filter, but that did not improve the prediction performance. There are also more sophisticated methods to classify time series data, for example through dynamic time warping [37] and hidden Markov models [38]. However, these methods are more complex and non-trivial to implement for predicting reactive stepping in real-time. As we could already obtain good results with a simpler, sample-based method we did not look further into classification methods for time-series.

This study had several limitations. First, we tried to predict natural balance responses in an experimental setting. It is not certain whether subjects indeed reacted naturally. By giving the subject the task to count backwards, we tried to ensure that they were not preparing a balance response. Still, in a few cases subjects seemed to react ‘unnaturally’ to a perturbation. By removing these data afterwards we tried to minimize the effects of unnatural balance responses. Second, the classification algorithms that we tested have hyperparameters, which we did not systematically optimize. By optimizing these hyperparameters to minimize the prediction error on the validation set, the performance of the classifiers could be further improved. Last, the classifiers were optimized for one specific dataset. The data in the training, validation, and test set were from the same subjects and the same experimental setting. Therefore, it is possible that the obtained results don’t generalize well to new subjects and other perturbations. Yet, we showed the potential of using a classification-based method for step prediction, and indicated important features for such a prediction.

### Possible applications & future work

In this work a classification-based method was presented to predict the occurrence of natural reactive stepping. Future work includes further validation of the results using data sets with other subjects and other perturbation types.

The classifiers obtained with the proposed method can be used in several applications, such as balance training and robotic support. In these cases, the trained classifiers can be implemented to decide whether or not the subject and/or robot should take or support a step to maintain balance. Therefore, the features need to be tracked real-time in various settings, inside and outside a biomechanics lab.

The setting of the application affects which feature set can be used. The kinematic features can be estimated using inertial measurement units on the body, or in case of robotic support, using joint encoders on the device. However, CoP-related measures are generally difficult to collect reliably outside a biomechanics lab. Therefore, if the application is not in a lab setting, within this method a classifier can be chosen that only requires kinematic input data to accurately predict reactive stepping.

Considering the step timing, given that an actual step has to be taken, the classifier should be able to make an early prediction. Hence a classifier that is trained using observations up until the moment of step leg unloading can be used for the prediction. A step can then for example be initiated when a new observation is classified as stepping, but it is also possible to wait until multiple samples predict a stepping response. The latter increases the robustness of the step prediction, but also increases the risk of initiating the step too late.

Ultimately, our goal is to implement this step prediction method in a human-like balance controller for an exoskeleton. Therefore, future research directions are to investigate the influence of the exoskeleton on reactive stepping, the timing of step initiation with respect to the timing of the step prediction and where to place the foot when a step is initiated.

## Conclusion

In this study we trained classification algorithms to predict natural reactive stepping in response to an external perturbation. We found that using a neural network with one hidden layer prediction accuracies could be obtained of 0.91 for predictions made up until the moment of step leg unloading and 1.00 for predictions made up until toe-off. In general, the performances were dependent on the timing of the step prediction - the later the prediction the higher the accuracy - and the feature set that was used for training the algorithms. The three most important features were the acceleration of the center of mass, the location of the cervical joint center and the velocity of the center of mass.

## Data Availability

The datasets generated and analysed during the current study are available in the 4TU repository, DOI: 10.4121/uuid:21cb6675-9b97-438b-b6d7-93313e27646b (data upload in progress)

## Abbreviations

BoS: Base of support
CJC: Cervical joint center
CoM: Center of mass
CoP: Center of pressure
LDA: Linear discriminant analysis
LSVM: Linear support vector machine
NN: Neural network
QDA: Quadratic discriminant analysis
RFE: Recursive feature elimination
SB: Stability boundary
SLU: Step leg unloading
TO: Toe-off
TTB: Center of mass-time-to-boundary
XCoM: eXtrapolated center of mass
